# Modeling [^18^F]-FDG lymphoid tissue kinetics to characterize nonhuman primate immune response to Middle East respiratory syndrome-coronavirus aerosol challenge

**DOI:** 10.1186/s13550-015-0143-x

**Published:** 2015-11-16

**Authors:** Svetlana Chefer, David Thomasson, Jurgen Seidel, Richard C. Reba, J. Kyle Bohannon, Mathew G. Lackemeyer, Chris Bartos, Philip J. Sayre, Laura Bollinger, Lisa E. Hensley, Peter B. Jahrling, Reed F. Johnson

**Affiliations:** Integrated Research Facility, Division of Clinical Research, National Institute of Allergy and Infectious Diseases, National Institutes of Health, Frederick, MD USA; Center for Infectious Disease Imaging, Radiology and Imaging Sciences, Clinical Center, National Institutes of Health, Bethesda, MD USA; Emerging Viral Pathogens Section, Division of Intramural Research, National Institute of Allergy and Infectious Diseases, National Institutes of Health, Frederick, MD USA; Present address: Visiting Scientist, Integrated Research Facility, Division of Clinical Research, National Institute of Allergy and Infectious Diseases, National Institutes of Health, Frederick, MD USA

**Keywords:** [^18^F]-FDG-PET, MERS-CoV, Animal models, Kinetic modeling

## Abstract

**Background:**

The pathogenesis and immune response to Middle East respiratory syndrome (MERS) caused by a recently discovered coronavirus, MERS-CoV, have not been fully characterized because a suitable animal model is currently not available. ^18^F-Fluorodeoxyglucose ([^18^F]-FDG)-positron emission tomography/computed tomography (PET/CT) as a longitudinal noninvasive approach can be beneficial in providing biomarkers for host immune response. [^18^F]-FDG uptake is increased in activated immune cells in response to virus entry and can be localized by PET imaging. We used [^18^F]-FDG-PET/CT to investigate the host response developing in nonhuman primates after MERS-CoV exposure and applied kinetic modeling to monitor the influx rate constant (*K*_*i*_) in responsive lymphoid tissue*.*

**Methods:**

Multiple [^18^F]-FDG-PET and CT images were acquired on a PET/CT clinical scanner modified to operate in a biosafety level 4 environment prior to and up to 29 days after MERS-CoV aerosol exposure. Time activity curves of various lymphoid tissues were reconstructed to follow the [^18^F]-FDG uptake for approximately 60 min (3,600 s). Image-derived input function was used to calculate *K*_*i*_ for lymphoid tissues by Patlak plot.

**Results:**

Two-way repeated measures analysis of variance revealed alterations in *K*_*i*_ that was associated with the time point (*p* < 0.001) after virus exposure and the location of lymphoid tissue (*p* = 0.0004). As revealed by a statistically significant interaction (*p* < 0.0001) between these two factors, the pattern of *K*_*i*_ changes over time differed between three locations but not between subjects. A distinguished pattern of statistically significant elevation in *K*_*i*_ was observed in mediastinal lymph nodes (LNs) that correlated to *K*_*i*_ changes in axillary LNs. Changes in LNs *K*_*i*_ were concurrent with elevations of monocytes in peripheral blood.

**Conclusions:**

[^18^F]-FDG-PET is able to detect subtle changes in host immune response to contain a subclinical virus infection. Full quantitative analysis is the preferred approach rather than semiquantitative analysis using standardized uptake value for detection of the immune response to the virus.

**Electronic supplementary material:**

The online version of this article (doi:10.1186/s13550-015-0143-x) contains supplementary material, which is available to authorized users.

## Background

The pathogenesis and immune response to Middle East respiratory syndrome (MERS) caused by a recently discovered coronavirus, MERS-CoV, has not been fully characterized, in part, because a suitable animal model that mimics human MERS is currently not available. Nonhuman primates (NHPs), such as rhesus monkeys (*Macaca mulatta*) or common marmosets (*Callithrix jacchus*) inoculated with MERS-CoV via combined intratracheal, intranasal, oral, and ocular routes, develop transient respiratory disease with little or no viremia although lethal disease was observed in a small number of marmosets [1–4]. ^18^F-Fluorodeoxyglucose ([^18^F]-FDG) PET/CT as a real-time noninvasive approach can be beneficial in providing biomarkers for host immune response and disease progression. [^18^F]-FDG-PET/CT has been used to track host immune response during monkeypox virus and human immunodeficiency virus-1 infections [5–7]. As [^18^F]-FDG uptake is increased in activated macrophages, lymphocytes, and granulocytes during inflammation, the immune response can be localized by PET imaging [8]. Tracking the host response noninvasively is especially useful when animal species studied is limited and/or expensive to obtain or when animals do not develop overt clinical signs of disease.

We applied [^18^F]-FDG-PET/CT imaging to monitor infection development in rhesus macaques after MERS-CoV inhalation. Compared to standardized uptake value (SUV), we increased the accuracy of measurement of [^18^F]-FDG uptake by applying kinetic modeling and Patlak graphical analysis. We assessed the net [^18^F]-FDG uptake rate constant (*K*_*i*_) in primary lymphoid tissues engaged in the host response to MERS-CoV exposure. This study is the first application of the methodology to an acute infectious disease process.

## Methods

### Ethics statement

Rhesus macaques were housed in a biosafety level 4 containment facility accredited by the Association for Assessment and Accreditation of Laboratory Animal Care International. Experimental procedures were approved by the National Institute of Allergy and Infectious Diseases (NIAID), Division of Clinical Research (DCR), Animal Care and Use Committee and were in compliance with the Animal Welfare Act regulations, Public Health Service policy, and the *Guide for the Care and Use of Laboratory Animals* recommendations.

### Virus preparation

For aerosol inhalation, MERS-CoV-Hu/Jordan-N3/2012 strain (GenBank accession no. KC776174.1) [9] was grown in Eagle’s Minimum Essential Medium (Lonza, MD, USA) on Vero E6 cells.

### Aerosol challenge

Prior to aerosol challenge, four rhesus macaques, two males and two females, 3–5 years old, weighing 3–5 kg each, were anesthetized by intramuscular ketamine (10–15 mg/kg) injection. Head-out plethysmography (Buxco-Data Sciences International, MN, USA) was used to calculate an average respiratory minute volume (mL/min) by multiplying the respiration rate by the tidal volume. Aerosol concentrations derived from a SKC biosampler (SKC Inc., PA, USA) were used to calculate the presented dose [10]. Within a negative-pressure (−24.9 Pa), head-only aerosol exposure chamber, macaques were exposed to a small-particle (0.5–3 μm aerodynamic diameter targeting lung alveoli) aerosol challenge (inhaled dose = log_10_ – 4.64 plaque-forming units).

### Data acquisition

Imaging data were acquired with Gemini PET/CT clinical scanner (Philips Healthcare, Andover, MA, USA) [5, 11]. With an axial field-of-view (FOV) of 180 mm of the PET scanner, the entire NHP thorax is imaged in a single bed position. Use of the scanner’s brain protocol resulted in a transverse field of view of 256 mm and led to cubic 2-mm-wide voxels in the reconstructed images. Low-dose CT images of the thorax for PET attenuation purposes were acquired at 120 kVp, 3-mm slice thickness, and 1.5-mm spacing. No contrast was given, and the subjects were freely breathing during the scan. PET image acquisition was initiated immediately after the low-dose CT scans and 1 min prior to intravenous injection of [^18^F]-FDG (9–10 MBq/kg) into the saphenous vein and continued for up to 60 min (3600 s). Nine imaging sessions per animal were conducted on pre-inoculation days −14 or −13 and −11 or −10 and post-inoculation days +1 or +2, +3 or +4, +7 or +8, +9 or +10, +15 or +16, +21 or +22, and +28 or +29 with MERS-CoV.

### Image reconstruction

SUV PET images were reconstructed iteratively using the manufacturer supplied 3D line-of-response (LOR)-based row-action maximum-likelihood algorithm [12]. Methods for scatter, decay, random, and attenuation correction were applied during the image reconstruction process. Both scatter and attenuation corrections [13] were based on the low-dose CT images acquired prior to the PET scans.

The list mode data were sorted into 46 dynamic frames during creation of the histograms. To extract the early tracer dynamic distribution in the arterial blood, the initial data set (up to 720 s or 12 min) was comprised of 39 frames with the following time sequence: 15 frames × 2 s, 6 frames × 5 s, 5 frames × 10 s, 5 frames × 20 s, 4 frames × 40 s, and 4 frames × 120 s. This sequence was followed by 3 frames × 240 s and 4 frames × 480 s to capture the late slow phase of dynamic distribution of the tracer in both the blood and the tissues. PET images were reconstructed iteratively using 3D ordered-subset expectation-maximization algorithm with two iterations and nine subsets followed by 18 iterations of maximum a posteriori reconstruction [14]. Maximum a posteriori parameters were adjusted to provide a uniform spatial resolution of 4.8 mm (full-width half-maximum = 4.8 mm) in all three directions. Methods for scatter, decay, random, and attenuation correction were applied during the process of PET image reconstruction.

### Volume of interest definition

**Fig. 1:**
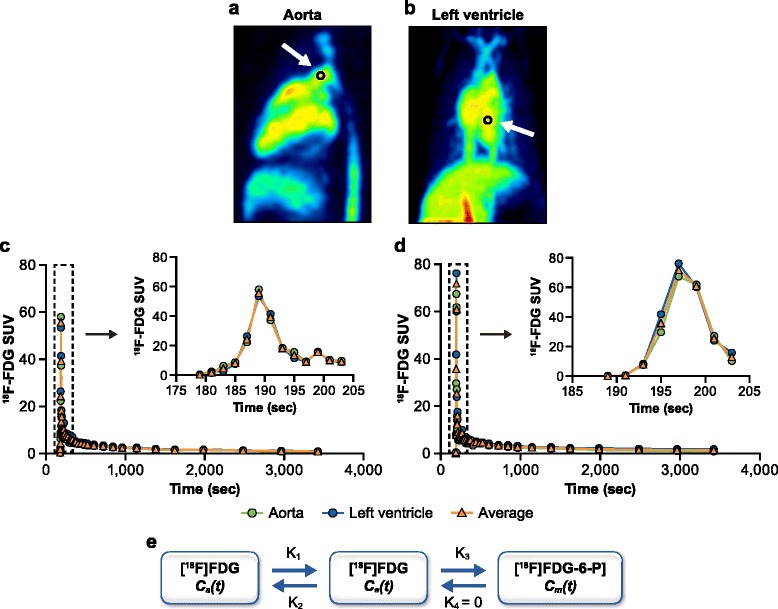
Measurement of image-derived input function. **a**, **b** SUV [^18^F]-FDG-PET images reconstructed 6 min (360 s) after [^18^F]-FDG bolus injection showing high concentrations of radiotracer in the arterial blood. *Black circles* on the images show the location of the VOIs, 2-mm spheres, in the arch of aorta, sagittal view (**a**), and the left ventricle, coronal view (**b**). **c**, **d** The kinetics of [^18^F]-FDG SUV in the arterial blood was determined in the left ventricle *(blue line)* and in the arch of aorta (*green line*), and the average of SUVs from these two sites is shown by the *orange line* in two animals. Inserts show SUVs from the subject and average of the two sites in the first 203 s after [^18^F]-FDG injection. **e** Two-tissue compartment kinetic model with irreversible tracer. *C*_*a*_(*t*) is the concentration of the tracer in arterial blood, *C*_*e*_(*t*) is the concentration of the tracer in extracellular space, and C_*m*_(*t*) is the concentration of the phosphorylated [^18^F]-FDG in the tissue. Abbreviations: FDG, fluorodeoxyglucose; VOI, volume of interest

Reconstructed SUV PET images were analyzed without any post-reconstruction smoothing using PMOD version 3.5 (PMOD Technologies, Zurich, CH). To extract an image-derived input function (IDIF), VOI (2-mm spheres) were placed on the left ventricles and arch of the aorta using frames over the first 6 min (360 s) after [^18^F]-FDG injection (Fig. 1a, b). Averaged data from two VOIs were used to generate the IDIF (Fig. 1c, d). Two-ml spheres were placed on axillary and mediastinal LNs and lumbar spine bone marrow as described previously [6], and 5-mm spheres were placed on right and left sides of the lungs to obtain the tissue time activity curves (TACs). The last time point of the TACs was used to generate the SUV data.

### Kinetic modeling

Using the standard two-tissue compartment kinetic model with irreversible tracer metabolism (*k*_4_ = 0, Fig. 1e), the tracer influx constant, *K*_*i*_, was computed for lymphoid and lung tissues [15]. The blood volume fraction (*V*_*b*_) was included in the modeling. Patlak linear regression method was applied for parameter estimation utilizing the IDIF, [^18^F]-FDG tissue TACs [16], and PMOD version 3.5 (PMOD Technologies). Tissue TACs were fitted to the models by use of the nonlinear least-squares method with the Levenberg-Marquardt algorithm, which minimizes the weighted sum of squared errors between PET measurement and model solutions. A plot of the ratio *C*_tis_(*t*)/*C*_bl_(*t*) against the ratio of cumulative to instantaneous blood activity concentration (“normalized time”) became linear in the late phase after the tracer injection when the concentration of free (i.e., unmetabolized) [^18^F]-FDG in the blood had equilibrated with that of free tracer in extravascular volume of distribution. This linear part of the plot was fitted by Eq. (1) to identify the *K*_*i*_ as a slope of a regression line:

1 $$ \frac{C_{\mathrm{tis}}}{C_{\mathrm{bl}}(t)}={K}_i\frac{{\displaystyle \underset{t=0}{\overset{t}{\int }}{C}_{\mathrm{bl}}(t)}}{C_{\mathrm{bl}}(t)}+{V}_{\mathrm{dist}} $$

in which *C*_tis_(*t*) and *C*_bl_(*t*) represent the radioactivity concentration in the region of interest and the arterial blood assessed from the PET images at different time points after an [^18^F]-FDG injection, respectively, and *V*_dist_ is an initial distribution volume. A criterion for maximum error was set to 5 % to derive the model parameter values. For the [^18^F]-FDG model described in Fig. 1, the slope equals *K*_1_ × *k*_3_ ÷ (*k*_2_ + *k*_3_).

### Hematology and clinical observations

Complete blood cell counts were determined on PET-scan days [5]. Body temperature or body weight were monitored once daily or once every other day, respectively.

### Statistical analysis

Two-way repeated measures analysis of variance (ANOVA) with post hoc Bonferroni multiple comparison test used *K*_*i*_ obtained pre-inoculation and post-inoculation with MERS-CoV and VOI location as independent variables to characterize the host immune response. For two-way repeated measures ANOVA, we used *K*_*i*_ at different time points pre-inoculation with MERS-CoV and VOI location as within and between two factors, respectively.

The correlations between *K*_*i*_ values in mediastinal and axillary LNs and bone marrow and between monocyte fraction in the blood and mediastinal and axillary LNs were calculated using the Pearson product moment correlation coefficient (*r*). The D’Agostino and Pearson test [17] was applied to confirm that the data followed a Gaussian distribution. GraphPad Prizm 6.01 (GraphPad Software Inc., La Jolla, CA, USA) was used for all statistical analyses.

## Results

### Host response to MERS-CoV challenge

**Fig. 2:**
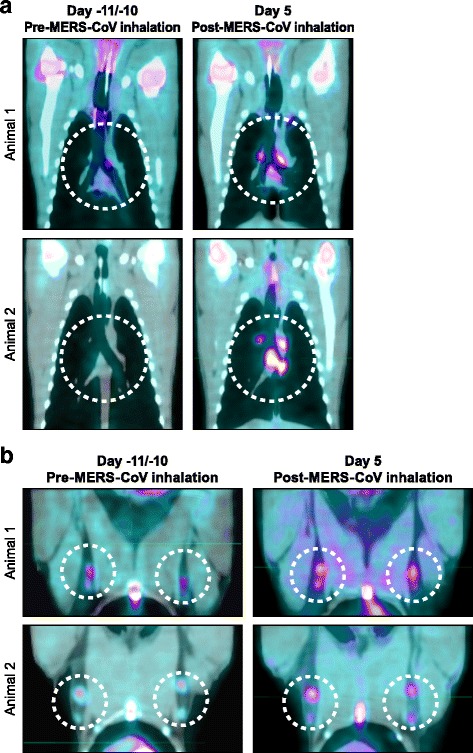
[^18^F]-FDG uptake in mediastinal (**a**) and axillary (**b**) LNs before and after MERS-CoV aerosol exposure in rhesus macaques. SUV [^18^F]-FDG-PET images coregistered with CT images showed tracer uptake in two representative rhesus macaques prior to and on day 5 after MERS-CoV aerosol exposure at 50–60 min after [^18^F]-FDG injection. *White dashed circles* show the location of the mediastinal and axillary LNs. Images are shown in coronal view. Abbreviations: FDG, fluorodeoxyglucose; LNs, lymph nodes; MERS-CoV, Middle East respiratory syndrome-coronavirus

**Fig. 3:**
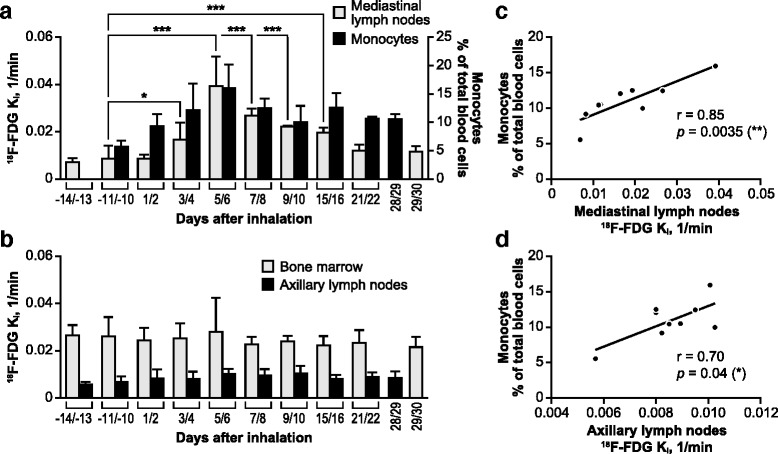
Changes in [^18^F]-FDG uptake and monocytes fraction in the blood after MERS-CoV aerosol challenge. **a** *K*_*i*_ in mediastinal LNs (*y*-axis on the *left*) and monocyte fraction in the blood (*y*-axis on the *right*) at different days prior to and after MERS-CoV exposure. **b** *K*_*i*_ in bone marrow and axillary LNs at different days prior to and after MERS-CoV exposure. Each column represents mean ± SD (*n* = 4). Post hoc analysis by Bonferroni’s multiple comparison test specified a statistically significant increase in *K*_*i*_ in mediastinal LNs up to days +15 or +16 post-exposure compared with the *K*_*i*_ values prior to virus challenge (adjusted *p* = 0.0152 on day +3 or +4, *p* < 0.0001 on days +5 or +6, +7 or +8, and +9 or +10, respectively, and *p* = 0.0004 on day +15 or +16). **c**, **d** Pearson product moment correlation between elevation in [^18^F]-FDG uptake in mediastinal (**c**) and axillary (**d**) LNs and changes in blood monocyte fraction. Abbreviations: FDG, fluorodeoxyglucose; *K*_*i*_, [^18^F]-FDG uptake rate constant; LNs, lymph nodes

Analysis of lung data revealed no pathology on CT images and no changes in [^18^F]-FDG uptake up to day 30 after MERS-CoV inhalation (data not shown). No changes in body temperature, body weight, and blood glucose concentrations (69.9 ± 7.4 mg/dL prior to exposure and 63.9 ± 10.4 mg/dL after exposure) were observed. However, [^18^F]-FDG uptake as indicated by SUV increases in mediastinal and axillary LNs post-inhalation (Fig. 2, Additional file 1: Movie S1). Analysis of complete blood cell counts revealed a slight increase (within normal range) in circulating monocytes only that peaked on day 5 or 6 post-inhalation and remained elevated through the remainder of the study (Fig. 3a).

### SUV time activity curves

**Fig. 4:**
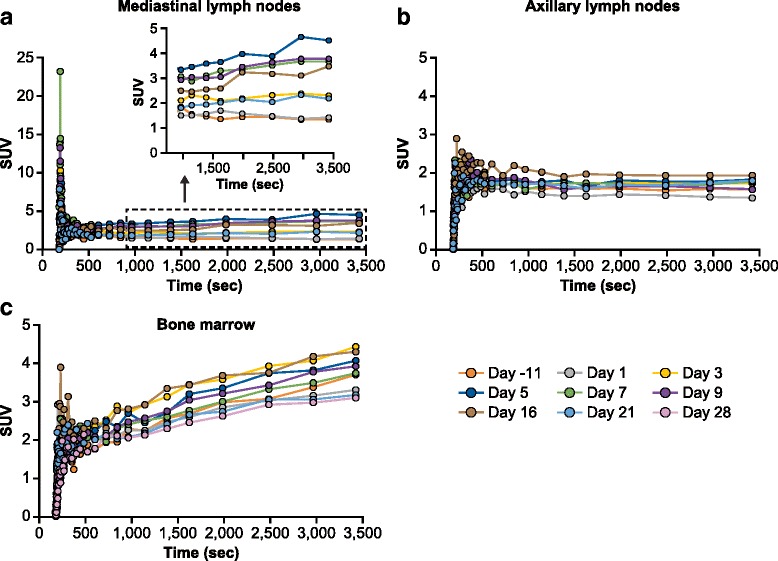
TACs of mediastinal LNs (**a**), axillary LNs (**b**), and bone marrow (**c**) prior to and after MERS-CoV aerosol exposure. Abbreviations: LNs, lymph nodes; MERS-CoV, Middle East respiratory syndrome-coronavirus; TAC, time activity curves

Images of the first 37 time frames comprised of 2–120 s each caught the fast kinetics of [^18^F]-FDG distribution in the arterial blood (Fig. 1c, d). The rest of six time frames, 4–8 min in duration, covered the slow distribution and accumulation of [^18^F]-FDG in the tissues at later time points (16–60 min, slow phase) (Additional file 2: Movie S2, Fig. 4). SUV TACs for axillary LNs plateaued 15 min (900 s) after FDG injection and were similar throughout the study duration of 1.5 months (Fig. 4b). Analogously, bone marrow SUV TACs did not show significant variation during the study. However, compared with the axillary LNs, the TACs for bone marrow continued to rise at 60 min (3,600 s) after [^18^F]-FDG injection (Fig. 4b, c) suggesting a longer time after [^18^F]-FDG injection for the tissue with high cell glycolytic activity to reach a steady state. In mediastinal LNs, TACs rise was pronounced on days 5–9 post-inhalation only, as indicated by TACs (Fig. 4a).

### Kinetic modeling

**Fig. 5:**
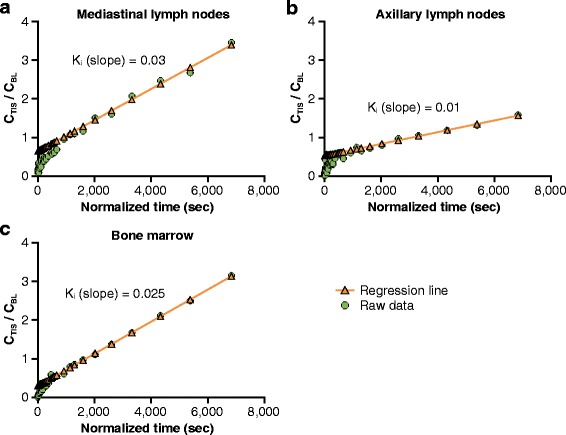
Patlak plots generated from IDIF and TACs from mediastinal (**a**) and axillary (**b**) LNs and bone marrow (**c**) on day 7 after MERS-CoV exposure in one representative animal. *Green dots* represent the experimental curve. Tissue [^18^F]-FDG net uptake rate (*K*_*i*_) is calculated as a slope of computed regression line (*orange triangles* and *solid lines*). For constant [^18^F]-FDG levels in arterial blood during the late phase, normalized time is identical to the true imaging time. Abbreviations: FDG, fluorodeoxyglucose; IDIF, image-derived input function; TACs, time activity curves

**Fig. 6:**
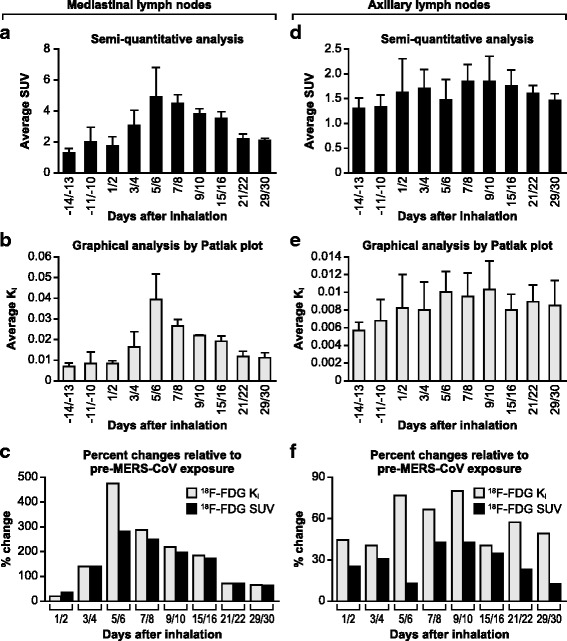
Host response assessed by Patlak graphical analysis and semiquantitative analysis using SUV. SUVs (**a**) and *K*_*i*_ (**b**) in mediastinal LNs. SUVs (**d**) and *K*_*i*_ (**e**) in axillary LNs. **c**, **f** Percent increase in SUV (*black columns*) and in *K*_*i*_ (*grey columns*) in mediastinal (**c**) and axillary (**f**) LNs was calculated as (*K*_*i*_ or SUV post-exposure) − (*K*_*i*_ or SUV pre-exposure) ÷ (*K*_*i*_ or SUV pre-exposure) × 100 %

Representative Patlak plots for bone marrow and mediastinal and axillary LNs are shown in Fig. 5. *K*_*i*_ obtained 2 days prior to virus exposure remained unchanged for each lymphoid tissue (Fig. 3a, b). The mean baseline *K*_*i*_ prior to MERS-CoV exposure was similar in axillary and mediastinal LNs (0.0062 ± 0.002 SD and 0.008 ± 0.004 SD, respectively). Greater elevation in mean *K*_*i*_ values (up to almost six-fold increase from pre-exposure scan) in mediastinal LNs was observed within the first week after MERS-CoV exposure compared to mean *K*_*i*_ values (<two-fold increase from pre-exposure scan) in axillary LNs (0.04 ± 0.01 SD and 0.01 ± 0.002 SD for mediastinal and axillary LNs on day 5/6 post-exposure), respectively, Figs. 3a, b and 6b, e). The [^18^F]-FDG mean *K*_*i*_ in bone marrow prior to MERS-CoV exposure was five-fold higher (0.03 ± 0.006 SD) compared to that observed in the LNs but did not follow the pattern of LN changes observed after virus challenge (Fig. 3b).

Statistical analysis using two-way repeated measures ANOVA revealed that alteration in *K*_*i*_ values was associated with the time point (*p* < 0.001) after virus exposure and the location of lymphoid tissue (*p* = 0.0004). As revealed by a statistically significant interaction (*p* < 0.0001) between these two factors, the pattern of changes in *K*_*i*_ over time differed between three locations but not between subjects. Post hoc analysis by Bonferroni’s multiple comparison test specified a statistically significant increase in *K*_*i*_ in mediastinal LNs post-exposure compared with the *K*_*i*_ values prior to virus challenge (*p* < 0.015, Fig. 3a). Correlation between *K*_*i*_ changes in axillary and mediastinal LNs was statistically significant, *r* = 0.758, *p* = 0.0179 (data not shown). In addition, changes in [^18^F]-FDG uptake as indicated by *K*_*i*_ in mediastinal and axillary LNs correlated with the elevation in percentage of monocytes in peripheral blood, *r* = 0.85, *p* = 0.0035 and *r* = 0.70, *p* = 0.04, respectively (Fig. 3c, d).

For comparison with the results obtained with *K*_*i*_, we evaluated the host response to MERS-CoV exposure using SUVs from 49 to 57 min (2940 to 3420 s) after [^18^F]-FDG injection (Additional file 3: Fig. S1c), the last time point from the TACs (Fig. 4a, b). The main difference between the findings using *K*_*i*_ and SUVs is that the correlations between the changes in mediastinal and axillary LNs (data not shown) and between monocytes fraction and the SUVs in axillary LNs were lost (Additional file 3: Fig. S1c). In addition, the first pronounced increase in [^18^F]-FDG uptake in axillary LN observed on day 5/6 post-exposure with *K*_*i*_ (Fig. 6e) was not detected when analyzed by SUVs (Fig. 6d). Furthermore, the magnitude of changes in axillary LNs associated with virus exposure was greater with *K*_*i*_ compared with SUVs, (Fig. 6c, f). For example, average percent change for *K*_*i*_ in axillary LNs was 52.9 ± 19.4 % SD, while an increase in [^18^F]-FDG uptake after MERS-CoV exposure determined by SUVs was about two-fold less, 25.1 ± 13.9 % SD.

## Discussion

We monitored ^18^F-FDG uptake primarily in lymphoid tissues at different locations in response to aerosol MERS-CoV challenge in NHPs. A group of mediastinal LNs showed a specific pattern of changes in *K*_*i*_ up to 29 days post-virus exposure that correlates to *K*_*i*_ changes in axillary lymph nodes. As the mediastinal LNs are the lung-draining LNs, these LNs had the greatest increase in *K*_*i*_ values compared to other lymphoid tissues. The increase in *K*_*i*_ in axillary LNs was 1/3 of that observed in mediastinal LNs and was not statistically significant. Nevertheless, changes in *K*_*i*_ in axillary and mediastinal LNs were concurrent with elevated percentage of monocytes in peripheral blood.

As shown by previously published studies, LN activation during viral infection generates an [^18^F]-FDG signal that can be easily discerned from background activity [7, 18]. Activated lymphocytes increase glucose uptake by approximately 20-fold over 24 h [19]. Moreover, results from a few longitudinal studies in rhesus macaques challenged with simian-human immunodeficiency virus or humans withdrawing from antiretroviral therapy indicate that increased LN tissue [^18^F]-FDG uptake preceded fulminant virus replication. Systemic factors such as viral proteins or cytokines could be engaged in lymphoid tissue response [7, 18]. As monocytes are part of the immune response to virus infection (e.g., cytokine release, phagocytosis), correlation between an increase in monocyte fraction and [^18^F]-FDG uptake in the LNs is not surprising.

We compared kinetic modeling using *K*_*i*_ to analysis using SUV to quantify [^18^F]-FDG uptake. By definition, SUV represents the radioactivity concentration in the region of interest adjusted for the injected dose and the body weight. Therefore, the SUV is influenced by several parameters such as (1) body composition, (2) blood glucose concentration, and (3) time interval from [^18^F]-FDG injection to image acquisition. As noted in TACs of bone marrow throughout the study and of mediastinal LNs on days 5–9 post-exposure, SUVs did not reach a plateau within 60 min (3600 s) after [^18^F]-FDG injection. Thus, high metabolic activity of proliferating cells in bone marrow requires longer time for [^18^F]-FDG to reach steady state in this tissue even under physiological conditions. As the LN biopsies have not been performed in the current study, future studies sampling the tissue from mediastinal LNs at peak response will be able to identify the specific cell type associated with increase in [^18^F]-FDG uptake in the LNs. Notably, by applying kinetic modeling analysis, we were able to determine a steady state parameter that characterizes the system based on two processes: [^18^F]-FDG delivery from the blood to the tissue of interest and the state of cell metabolic activity. As multiple time points from PET-acquired data are used in the kinetic modeling analysis, the accuracy of *K*_*i*_ computation for small regions of interest is higher compared with SUVs even in the presence of high noise [16]. In this study, kinetic modeling increases the signal-to-noise ratio resulting in an improvement of the accuracy of measurement and method sensitivity over analysis with SUV. If the effect is mild, then a semiquantitative analysis by SUV will not be able to detect it.

In this study, we attempted to develop an aerosol NHP model of MERS-CoV infection. The current NHP aerosol model failed to cause overt signs of MERS-CoV infection. Comparison of our results to other MERS-CoV challenge studies in rhesus macaques is hampered by the different dose units and route(s) of administration. Doses given in previous studies were expressed as tissue culture infectious dose 50 % (TCID_50_) and was given by a combination of intratracheal, ocular, oral, and intranasal routes [2, 3] or by intratracheal route only [4]. Nevertheless, as [^18^F]-FDG-PET quantitated the host response to virus challenge while CT revealed no pathology on the images, the sensitivity of CT for detection of host response is lower than [^18^F]-FDG-PET [20]. Similarly, results from a MERS-CoV exposure in rabbits demonstrated that MERS-CoV replicates in the lung tissue without the development of overt clinical signs [21]. As subclinical MERS-CoV infection has been reported more often in patients without comorbidities than in patients with comorbidities [22–24], this animal model can be used to study the host response to MERS-CoV and to test the intervention strategies aimed at inhibition of MERS-CoV replication [21]. However, suppression of immune responses to MERS-CoV exposure in these animals may be needed to reflect the severity of MERS observed in the majority of patients.

Other limitations of this feasibility study are the small number of subjects and preexisting health status. As this study used young, previously healthy animals with an apparently intact immune response, translation of these findings to older MERS patients with numerous comorbidities should be done with caution. Since [^18^F]-FDG-PET identifies the location and the pattern of host response to virus challenge, future studies with a higher challenge dose will investigate changes in [^18^F]-FDG uptake along with results of histopathological and immunological analysis of responsive lymphoid tissues.

[^18^F]-FDG is generally considered to be a nonspecific tracer for inflammation/immune activation, and the cells involved in the host response cannot be specified by [^18^F]-FDG-PET. However depending on the virus and the disease stage, certain populations of immune cells will be involved in the response. Therefore, the location and pattern of [^18^F]-FDG changes in the body during infection progression will be virus specific. Compared with clinical application in which [^18^F]-FDG-PET is mostly used for diagnostic purposes, preclinical studies are performed under controlled conditions using within subject design. The metabolic map of the body determined prior to virus exposure is used to identify the location of the tissues with elevated glycolytic activity after the exposure. The specific pattern of [^18^F]-FDG changes in the foci identified by PET at pre- and post-exposure states are monitored day-by-day. When the pattern and the location of [^18^F]-FDG changes are identified following virus exposure, this pattern can be used to monitor the effects of treatment. In our previous study [6], we described the host response to monkeypox virus showing the pattern and the location of [^18^F]-FDG changes in lymphoid tissues that was distinctive from that observed after MERS-CoV exposure. In addition, we were able to describe the difference between surviving and non-surviving animals that was detected at early stages of monkeypox infection. Further ongoing studies of the analysis of biopsied tissue samples from the locations identified by PET should be able to shed some light on specific mechanisms associated with [^18^F]-FDG changes after virus exposure.

## Conclusions

This study extends the use of kinetic modeling of [^18^F]-FDG uptake during lung inflammation to host immune response after MERS-CoV exposure in rhesus macaques. Compared with SUVs, *K*_*i*_ computation increases signal-to-noise ratio of PET data that results in improved accuracy of measurement and method sensitivity. With application of kinetic modeling of [^18^F]-FDG uptake in infected animals, researchers will be able to pinpoint sites of immune response and quantify anti-inflammatory effects of potential antiviral treatments.

### Statement on welfare of animals

Rhesus macaques were housed in a biosafety level 4 containment facility accredited by the Association for Assessment and Accreditation of Laboratory Animal Care International. Experimental procedures were approved by the National Institute of Allergy and Infectious Diseases (NIAID), Division of Clinical Research (DCR), Animal Care and Use Committee and were in compliance with the Animal Welfare Act regulations, Public Health Service policy, and the *Guide for the Care and Use of Laboratory Animals* recommendations.

## Additional files

Additional file 1: Movie S1. Maximum intensity projection movie showing maximal [^18^F]-FDG uptake in mediastinal LNs in 3-D in one representative animal on day 5 after MERS-CoV exposure. Abbreviations: FDG, fluorodeoxyglucose; LNs, lymph nodes; MERS-CoV, Middle East respiratory syndrome-coronavirus. (MP4 707 kb) Additional file 2: Movie S2. Distribution and clearance of [^18^F]-FDG in the body measured via SUV. Dynamic images were taken over 60 min after [^18^F]-FDG injection on day 5 after MERS-CoV exposure in one representative animal. Each image in a series represents a sum of [^18^F]-FDG concentrations in three compartments defined by a two-tissue compartment model. The first 37 images demonstrate the early fast [^18^F]-FDG distribution and clearance from the blood, while the next 7 images capture the late slow phase of dynamic [^18^F]-FDG distribution in the blood and accumulation of [^18^F]-FDG-6-P in the mediastinal LNs. Abbreviations: FDG-6-P, fluorodeoxyglucose-6-phosphate; LNs, lymph nodes; MERS-CoV, Middle East respiratory syndrome-coronavirus. (MP4 837 kb) Additional file 3: Figure S1. Changes in [^18^F]-FDG uptake and monocytes fraction in the blood after MERS-CoV aerosol challenge. (a) SUVs in mediastinal LNs (*y*-axis on the left) and monocyte fraction in the blood (*y*-axis on the right) at different days prior to and after MERS-CoV exposure. Each column represents mean ± SD (*n* = 4). (b) SUVs in bone marrow and axillary LNs on different days prior to and after MERS-CoV exposure. Post hoc analysis by Bonferroni’s multiple comparison test specified a statistically significant increase in SUV in mediastinal LNs up to days +14 or +15 post-exposure compared with the SUVs prior to virus challenge (adjusted *p* = 0.0006 on day +3 or +4, *p* < 0.0001 on days +5 or +6, +7 or +8, +9 or +10, and +15 or +16 respectively). (c, d) Pearson product moment correlation between elevation in [^18^F]-FDG uptake in mediastinal (c) and axillary (d) LNs determined by SUV and changes in blood monocyte fraction. Note that the correlation between SUV in axillary LNs and monocyte fraction lost statistical significance. Abbreviations: FDG, fluorodeoxyglucose; *K*_*i*_, [^18^F]-FDG uptake rate constant; LNs, lymph nodes. (PDF 206 kb)
