# Supplementary figures and images for: Modeling [18F]-FDG lymphoid tissue kinetics to characterize nonhuman primate immune response to Middle East respiratory syndrome-coronavirus aerosol challenge
Source: EJNMMI Res. 2015 Nov 16;5:65. doi: 10.1186/s13550-015-0143-x (PMC4646887; doi:10.1186/s13550-015-0143-x)

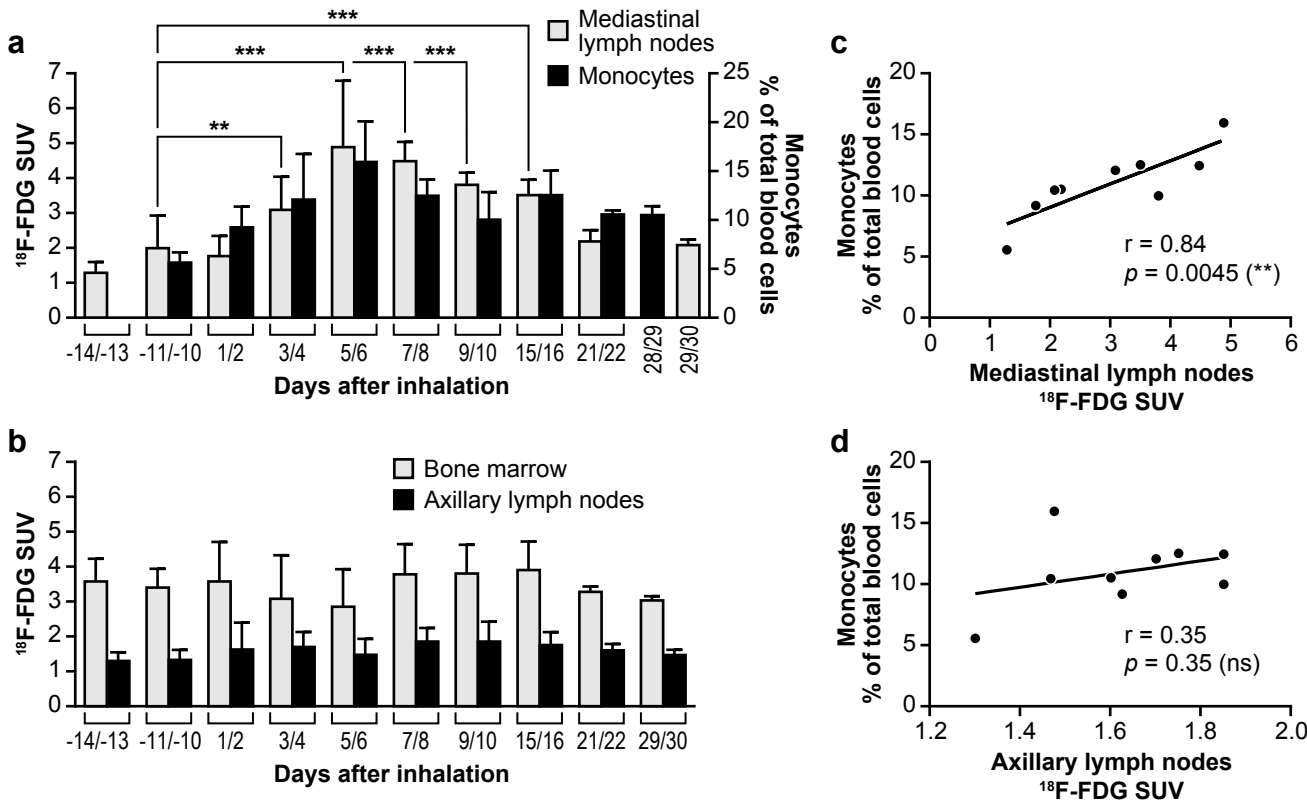

Supplement: Additional file 3: Figure S1. — Changes in [18F]-FDG uptake and monocytes fraction in the blood after MERS-CoV aerosol challenge. (a) SUVs in mediastinal LNs (y-axis on the left) and monocyte fraction in the blood (y-axis on the right) at different days prior to and after MERS-CoV exposure. Each column represents mean ± SD (n = 4). (b) SUVs in bone marrow and axillary LNs on different days prior to and after MERS-CoV exposure. Post hoc analysis by Bonferroni’s multiple comparison test specified a statistically significant increase in SUV in mediastinal LNs up to days +14 or +15 post-exposure compared with the SUVs prior to virus challenge (adjusted p = 0.0006 on day +3 or +4, p < 0.0001 on days +5 or +6, +7 or +8, +9 or +10, and +15 or +16 respectively). (c, d) Pearson product moment correlation between elevation in [18F]-FDG uptake in mediastinal (c) and axillary (d) LNs determined by SUV and changes in blood monocyte fraction. Note that the correlation between SUV in axillary LNs and monocyte fraction lost statistical significance. Abbreviations: FDG, fluorodeoxyglucose; K i, [18F]-FDG uptake rate constant; LNs, lymph nodes. (PDF 206 kb) [file 13550_2015_143_MOESM3_ESM.pdf]
